# Patient-Reported Measurement of Breast Asymmetry Using Archimedes’ Principle in Breast Reduction Mammaplasty: A Retrospective Study

**DOI:** 10.7759/cureus.6536

**Published:** 2020-01-01

**Authors:** Daniel Waltho, Mark McRae, Achilleas Thoma

**Affiliations:** 1 Plastic Surgery, McMaster University, Hamilton, CAN

**Keywords:** breast, reduction, mammoplasty, asymmetry, archimedes, plastic surgery, measurement, patient-reported, displacement

## Abstract

Introduction

Breast hypertrophy is a common condition that is often treated with breast reduction surgery. A large percentage of breast hypertrophy patients have notable asymmetry between breasts.

Methods

The purpose of this study was to investigate a method of measuring breast asymmetry, one that allows patients to determine the asymmetry of their own breasts at home with ease, and to assess its accuracy and role in a surgical practice. A retrospective chart review was conducted, wherein self-measurements of breast asymmetry using a variation of Bouman’s technique were compared with the recorded intra-operative resected tissue mass.

Results

In total, 47 patients with asymmetry were included in the study. The difference between patient-reported measurements and resected breast tissue mass varied from 0 grams to 240 grams. Of the 47 patients, 38% were able to measure their breast difference within a remarkable 10 grams as compared to the resected breast tissue, of which four patients were accurate to less than one gram. The majority (70%) of patients accurately measured their asymmetry within 50 grams, which was determined to be a clinically significant amount based on a survey of plastic surgeons performed for the study.

Conclusion

The breast measurement technique presented in this study appears to be effective and accurate for most patients with suspected asymmetry undergoing reduction mammaplasty that stands to reduce pre-operative planning time. Patient-reported breast measurement may emerge as a valuable tool in clinical and research pursuits; however, further research on this topic is indicated at this time.

## Introduction

Breast hypertrophy (i.e., macromastia or gigantomastia) is a common condition seen by plastic surgeons [1]. Patients with breast hypertrophy often suffer from back and shoulder pain, intertrigo of the inframammary fold, and shoulder grooving due to brassiere straps [1]. Moreover, studies show that breast hypertrophy is associated with significant negative impacts on a woman’s health status and quality of life [2]. The mainstay of treatment for breast hypertrophy is breast reduction, which is one of the commonest procedures performed in plastic surgery [3]. In reduction mammaplasty, one removes breast tissue (skin, adipose, glandular) and transposes the nipple-areolar complex for the purposes of smaller, symmetrical, and cosmetically appealing breasts. Breast reduction has been shown to significantly improve patient satisfaction, including breast appearance as well as the physical, psychological, and sexual well-being of the patient [4], and overall improve the quality of life of the patient [5]. As alluded to already, breast symmetry is one of the primary goals of this procedure, particularly in terms of producing aesthetically pleasing breasts and even mass distribution between the left and right breasts. In many cases, marked breast asymmetry may already exist pre-operatively. In one study, 20% of breast hypertrophy patients were found to have a difference of greater than 200 grams between breasts [6]. For some patients, their asymmetry may go unnoticed until pre-operative examination, while for others, it may be guiding their decision for surgery. It is thus important for surgeons to be aware of the extent of asymmetry prior to operating to ensure optimal results.

Several methods exist to measure breast size and diagnose asymmetry that avoids unnecessary imaging. Anthropometric measurements are often performed to some degree by the plastic surgeon pre-operatively and post-operatively. In this method, surgeons use anatomical landmarks on the patient to identify breast dimension changes before and after the procedure, as well as between-breast differences; anthropometric measurements can also be used to estimate breast volume [7]. Another method uses a Grossman-Roudner device, which is a graduated disc that can be folded into a conical shape to measure volume [7]. Furthermore, breast casts can be made out of a moldable material, and volume can then be determined by filling the cast with water [7]. Three-dimensional photography has also been shown to be comparable with other breast measurement techniques but requires expensive hardware and software [8]. A final method, and the topic of this study, uses Archimedes’ principle of water displacement. In this method, the patient’s breast is submerged in water and the displaced water is measured, which is equal to the volume of the breast [9]. Archimedes’ principle has been applied in a variety of techniques for measuring breast volume; however, the one focused on in this study was first described by Bouman [9]. With Bouman’s technique, the patient bends forward over a large container brimming with water and submerges her breast until the brim of the container is touching her chest wall around the entire breast [9]. The volume of water required to refill the container is thus equal to the breast measurement [9]. This technique separates itself from other methods of breast measurement in that it requires no anatomical knowledge or any specific clinical equipment. While the existing literature using Archimedes’ principle involved measurements that were performed in the clinic or operating room (OR), we believe that the patient can effectively and accurately perform this technique outside of the clinic and without the presence of a medical professional.

The purpose of this study is to determine if breast reduction patients can accurately measure their breast volume to identify their degree of breast volume asymmetry. We present a series of cases, wherein patients self-measured their breasts prior to reduction mammaplasty surgery using a variation of Bouman’s technique. By comparing the difference in patient-reported volume between the right and left breasts with the respective resected tissue mass to achieve symmetry intra-operatively, we assess the accuracy of patient-performed measurement results.

## Materials and methods

Study design and study population

This is a retrospective study. The setting of the surgeries is an academic hospital. The clinical records of all patients who underwent reduction mammaplasty (government approved) between March 20, 2012, and August 1, 2015, by the senior author (Achilleas Thoma) at St. Joseph’s Healthcare in Hamilton, Ontario, Canada, were searched to identify patients who had provided breast volume measurements based on a specific instruction detailed below. All patients who were asked to measure their breast volumes and successfully did so were included in the study.

The pre-operative volume asymmetry measured by the patient was correlated with the resected tissue masses that had been weighed in the OR prior to being sent to pathology. Determination of the amount of tissue resected in each case was decided by the surgeon, with input from the surgical assistants and nursing staff. Documented follow-up of both patient and surgeon satisfaction with post-operative symmetry was taken as a sign that the resected mass had achieved the desired symmetry.

Data extraction and analysis

Data on patient breast measurements were collected from the patients’ health file, along with pathology records of the mass of breast tissue resected at the time of surgery. For patient-reported breast measurements, all recorded measurements were included in the analysis by determining the mean of measurements within each breast. Data on patients’ age, height, mass, and brassiere measurement were also collected.

Differences in volume (mL) between the patient’s right and left breasts were converted to mass (grams) based on the conversion of water (1 mL = 1 gram). The difference between patient-reported measurements of left and right breasts was compared with that of the respective resected tissue samples to ultimately calculate the difference between “patient asymmetry” and “true asymmetry”.

Breast volume measurement method

**Figure 1:**
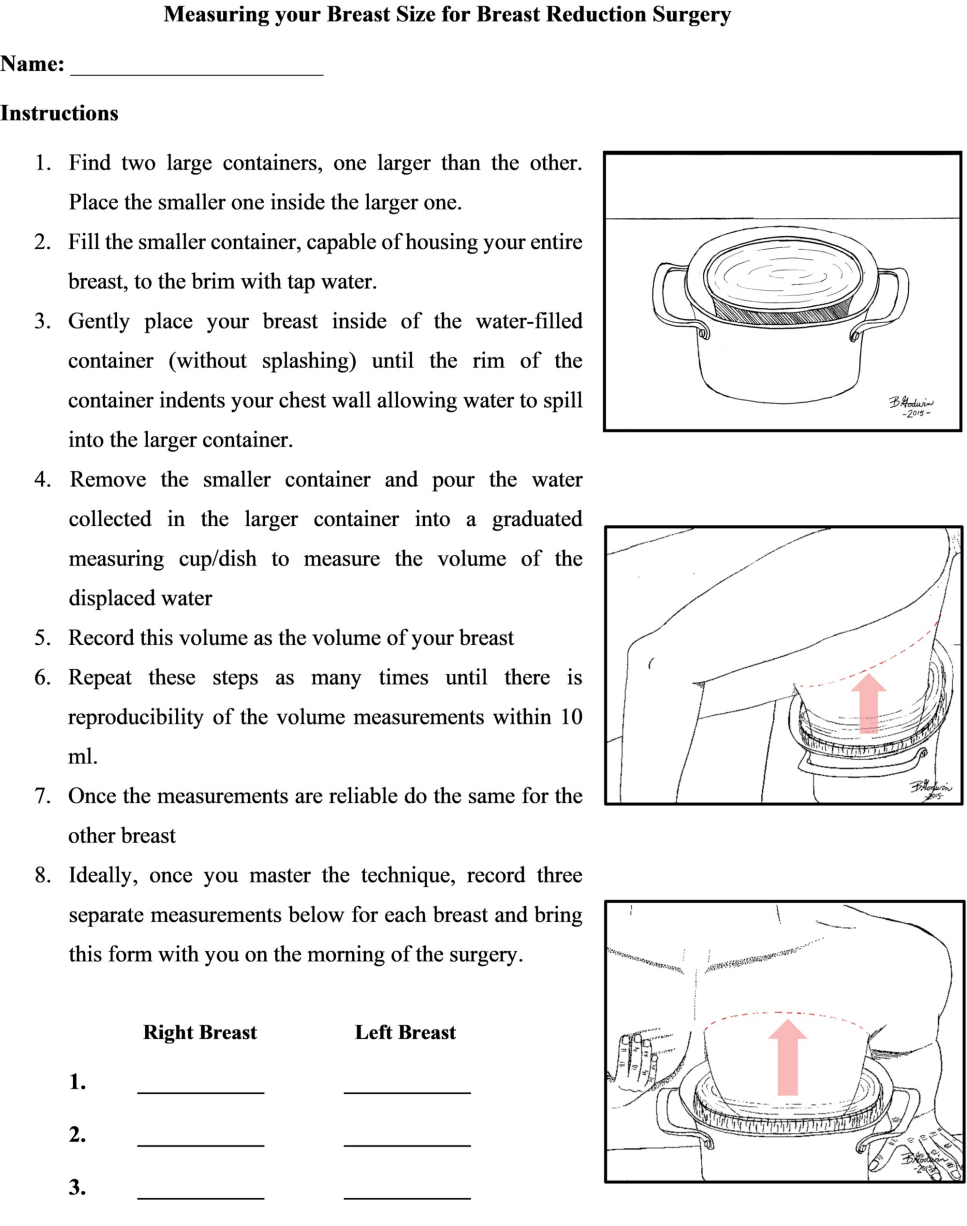
Patient handout for breast measurement technique

During pre-operative consultation with the plastic surgeon (Achilleas Thoma), patients were provided with step-by-step instructions on how to measure the volume of their breasts, including a written diagram for them to take home. The instructions are detailed in the patient handout shown in Figure 1.

## Results

Baseline cohort characteristics

Between March 20, 2012, and August 1, 2015, a total of 127 patients underwent reduction mammaplasty. Of these, 47 patients were found to have noticeable asymmetry by themselves and the surgeon.

This subset of 47 patients completed the breast measurement protocol and recorded at least one measurement for each breast. The mean age of the patients was 44.62 years (range: 20-71 years), mean mass was 76.32 kg (range: 28.58-117.94 kg), mean height was 64.13 inches (range: 59-71 inches), and mean BMI was 28.74 (range: 11.16-46.05) (Table 1). The brassiere size ranged from D to K (patient-reported). All reduction mammaplasties were performed using the vertical scar technique.

**Table 1:** Patient demographic information

Demographic		No. of patients	Percent
Total		47	
Age (years)			
Mean	44.62		
Range	20-71		
Brassiere size			
D		4	8.51%
DD		18	38.30%
DDD		4	8.51%
E		2	4.26%
EE		1	2.13%
E/F		1	2.13%
F		5	10.64%
G		6	12.77%
GG		1	2.13%
H		1	2.13%
I		2	4.26%
J		1	2.13%
K		1	2.13%
Height (inches)			
Mean	64.13		
Min, max	59, 71		
Mass (kg)			
Mean	76.32		
Min, max	28.58, 117.94		
BMI			
Mean	28.74		
Min, max	11.16, 46.05		

Relationship between patient measurements and resected tissue

Table 2 summarizes all reduction mammaplasty cases and the respective measurements of breast volume asymmetry.

**Table 2:** Summary of results

Patient no.	Resected tissue (g)			Patient-reported (g)			Patient difference vs. resected tissue difference (patient – resected tissue)
	Right breast	Left breast	Difference (left – right)	Right breast	Left breast	Difference (left – right)	
1	525	470	-55	1079.17	1020.00	-59.17	4.17
2	840	585	-255	2000.00	1700.00	-300.00	45.00
3	350	435	85	656.67	756.67	100.00	15.00
4	400	450	50	384.67	441.67	57.00	7.00
5	790	640	-150	2100.00	1950.00	-150.00	0.00
6	325	400	75	800.00	875.00	75.00	0.00
7	310	340	30	640.00	650.00	10.00	20.00
8	203	340	137	390.00	560.00	170.00	33.00
9	340	490	150	850.00	983.33	133.33	16.67
10	290	400	110	887.50	975.00	87.50	22.50
11	310	410	100	561.67	663.33	101.67	1.67
12	430	440	10	500.00	500.00	0.00	10.00
13	540	520	-20	1656.00	1636.33	-19.67	0.33
14	450	490	40	1000.00	962.50	-37.50	77.50
15	250	230	-20	414.00	369.75	-44.25	24.25
16	450	550	100	2123.33	2252.67	129.33	29.33
17	700	620	-80	1985.00	1720.00	-265.00	185.00
18	525	340	-185	1400.00	1100.00	-300.00	115.00
19	435	615	180	635.67	818.17	182.50	2.50
20	290	210	-80	1037.50	950.00	-87.50	7.50
21	710	520	-190	2313.33	2113.33	-200.00	10.00
22	315	265	-50	987.50	966.67	-20.83	29.17
23	305	210	-95	858.33	700.00	-158.33	63.33
24	300	300	0	597.00	610.67	13.67	13.67
25	300	310	10	525.00	533.33	8.33	1.67
26	410	340	-70	893.33	833.33	-60.00	10.00
27	340	420	80	725.00	810.00	85.00	5.00
28	220	200	-20	443.33	433.33	-10.00	10.00
29	230	330	100	560.00	658.33	98.33	1.67
30	350	360	10	985.00	816.67	-168.33	178.33
31	330	440	110	875.00	1026.04	151.04	41.04
32	450	435	-15	180.00	246.67	66.67	81.67
33	500	570	70	1756.33	1828.33	72.00	2.00
34	315	540	225	1060.00	1202.50	142.50	82.50
35	315	285	-30	786.67	755.33	-31.33	1.33
36	300	430	130	1409.33	1509.00	99.67	30.33
37	440	580	140	1600.00	1822.92	222.92	82.92
38	960	1150	190	2041.67	2150.00	108.33	81.67
39	730	900	170	1100.00	1250.00	150.00	20.00
40	410	570	160	900.00	1091.67	191.67	31.67
41	390	380	-10	766.67	516.67	-250.00	240.00
42	300	400	100	963.00	1188.00	225.00	125.00
43	460	670	210	495.00	636.67	141.67	68.33
44	265	350	85	1080.67	1152.00	71.33	13.67
45	180	180	0	383.33	283.33	-100.00	100.00
46	480	500	20	821.67	771.67	-50.00	70.00
47	370	320	-50	1341.67	1291.67	-50.00	0.00

The difference between patient-reported measurements and resected breast tissue mass varied from 0 grams to 240 grams. There is no association between discrepancies within patient-reported measurements and the original breast size. Of the 47 patients, 26 (55%) underestimated their breast volume asymmetry, whereas 18 (38%) overestimated it.

**Figure 2:**
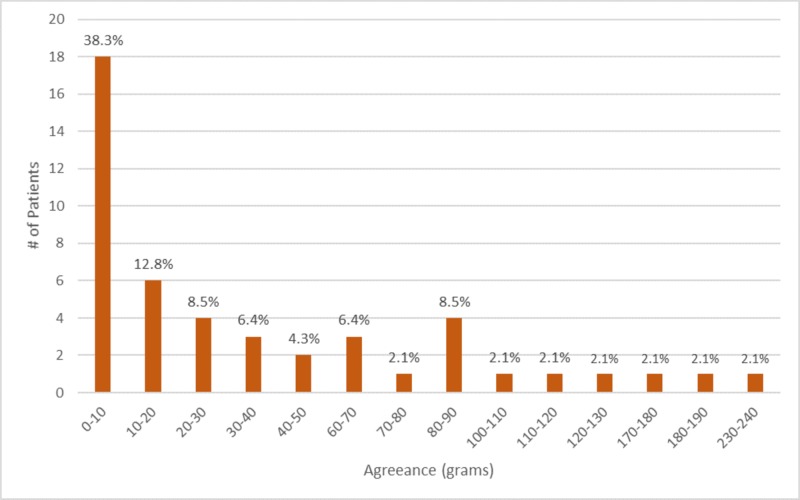
Magnitude of difference between patient-measured asymmetry and resected tissue asymmetry

Of the 47 patients, 38% were able to measure their breast difference within 10 grams of the resected breast tissue, of which four patients were accurate to less than one gram. The majority (70%) of patients accurately measured their asymmetry within 50 grams. Figure 2 summarizes the proportion of agreement between all patient-measured breast volume asymmetry and asymmetry determined by the resected tissue.

## Discussion

Archimedes, the great Greek mathematician, would have been amused by the application of his principle in breast reduction. Although his head was on the line if he had not solved the problem of whether the gold ring was indeed pure gold and not alloy [10], the plastic surgeons’ fate today is much safer even if we fail to achieve symmetry of the breasts.

This review compares breast volume asymmetry measured by patients with “true breast volume asymmetry” determined by the resected tissue mass in reduction mammaplasty. A survey of nine plastic surgeons within the Hamilton Health Sciences and the city of Ottawa, Ontario, Canada, was conducted to determine the smallest difference in weight between the left and right breasts that would allow them to identify breast volume asymmetry on inspection. The results of this survey revealed an average of 47 grams, which can be considered the minimal clinically important difference for “observable” asymmetry. Of the patients, 70% were able to measure their breast volume asymmetry to an accuracy of at least 47 grams, meaning the large majority of patients were able to provide measurements that are clinically important.

In six cases, however, patient measurements were exceedingly different from that of resected tissue measurements (>100 grams [mL]). One patient had a breast measurement that was over 200 grams (mL). The reason for these discrepancies is likely attributed to improper measurement technique of the patient. These observations suggest that the method of patient-reported breast measurement may not be appropriate for all patients. There were no apparent demographic patterns, including breast size and patient BMI, to explain those patients who did not adequately measure their breast volume asymmetry, nor were there any methodological differences. A prospective study on this topic may reveal specific population factors that influence the success of breast measurement.

Our study has some inherent limitations. Although 1 mL of water is equal to 1 gram, the density of a resected tissue sample may not be exactly equal to that of water, thus skewing the conversion between patient measurement volumes and resected tissue mass. Moreover, minimal asymmetry may have still existed, but gone undetected, in the post-operative breasts due to the qualitative assessment used, which may have led to miscalculations of the original true asymmetry. Furthermore, as this is a retrospective study, we could not investigate all plausible variables, in particular identifying the population for which this tool is appropriate. Finally, nuances between patients’ chest walls, skeletal framework (i.e., scoliosis), and degree of breast ptosis may influence the results of their breast measurement but were not able to be directly studied due to the retrospective design. Socioeconomic status and education level would have been interesting variables to consider in those cases in which there was a large discrepancy between “self-report volume” and resected tissue.

Good communication and written instructions for the patient to take home with them is the key to accurate results. Figure 1 may be used as a one-page handout for the patient.

## Conclusions

In conclusion, our study offers evidence that this method of breast measurement can be a helpful tool for patients with suspected asymmetry undergoing reduction mammaplasty. Use of this method not only engages the patients in their surgical care but also provides surgeons with information on the magnitude of breast volume asymmetry prior to the surgical procedure. Additional prospective research is indicated moving forward to further validate this method of patient-reported breast measurement and to identify the appropriate population for this measurement.
